# Integrating Health Care Delivery and Data Collection in Rural India Using a Rapidly Deployable eHealth Center

**DOI:** 10.1371/journal.pmed.1001468

**Published:** 2013-06-25

**Authors:** Anurag Agrawal, Jaijit Bhattacharya, Nishant Baranwal, Sushil Bhatla, Salil Dube, Viren Sardana, Devender R. Gaur, Danica Balazova, Samir K. Brahmachari

**Affiliations:** 1 Council of Scientific and Industrial Research (CSIR), India - Institute of Genomics and Integrative Biology; 2 Hewlett Packard, India; 3 OP Jindal Gramin Jan Kalyan Sansthan (OP Jindal Rural Welfare Society); 4 Maharaj Agrasen Medical College (MAMC), Agroha, India; 5 Council of Scientific and Industrial Research (CSIR), India – Central Scientific Instruments Organization

## Abstract

Anurag Agrawal and colleagues describe their experience of setting up a readily deployable cargo container-based health center in rural India.

*Please see later in the article for the Editors' Summary*

**Summary Points**

- Creation of functional health infrastructure in developing countries is difficult; lag time is long, adequate installation is unverified, on-site medical manpower is scarce, and quality of health care is difficult to assess.
- Health care planning as well as the monitoring and evaluation of interventions are hindered by a lack of records.
- An integrated rapidly deployable solution, the eHealth Center, was created in cargo containers, with verifiable cloud-based electronic workflow and records, telemedicine capability, and automated online reporting of summarized health data and operational status.
- We report the experience and learning from the first such installation, which occurred in a small Indian village.

## The Challenge

Inequity in health care access is a major cause of increasing disease burden, catastrophic medical expenses, and inability to escape poverty, especially in the developing world [1]–[6]. Government programs to effect disease surveillance and treatment do exist, such as for tuberculosis, yet proper implementation is often lacking, especially in rural regions [1],[5]–[7]. The foremost limitation is a real shortage of health care providers coupled with inequitable concentration of resources, of both manpower and infrastructure. This limitation is amplified by an inability to monitor the use of the few resources that are available, diversion of funds earmarked for health infrastructure creation, and provider absenteeism. The general lack of objective health data makes informed or targeted disease prevention difficult, especially in the context of limited resources [8].

Attempts to bridge the health care gap through telemedicine have met with limited success [9],[10] because of a fundamental lack of infrastructure and transparency in operations. The challenge is to create a rapidly deployable infrastructure with transparent data-driven operations that integrates the process of healthcare delivery and healthcare data collection. The initial concepts of “doc-in-a-box", where cargo-shipping containers are utilized for rapidly deploying a health care infrastructure, have already been provided by Garret [11]. Such rapidly deployable units have been used effectively by global health organizations, such as by Medicine-sans-Frontiers in the aftermath of the 2010 earthquake in Haiti and by Containers2Clinics for maternal-child welfare programs [12],^13^. Yet the health care community lacks an integrated solution that harnesses together the infrastructure-creation advantages of cargo containers; the capability of telemedicine to enable high-quality healthcare access; operational transparency of a cloud-based electronic workflow; and automated analysis of data for various levels of decision support [14]. In this *Health in Action*, we report the proof-of-concept for an integrated solution that is centrally fabricated, easily deployable, telemedicine-capable with provision for decision-support, and easy to monitor.

## An Integrated eHealth Center (eHC)

### eHC Structure and Infrastructure

**Figure 1:**
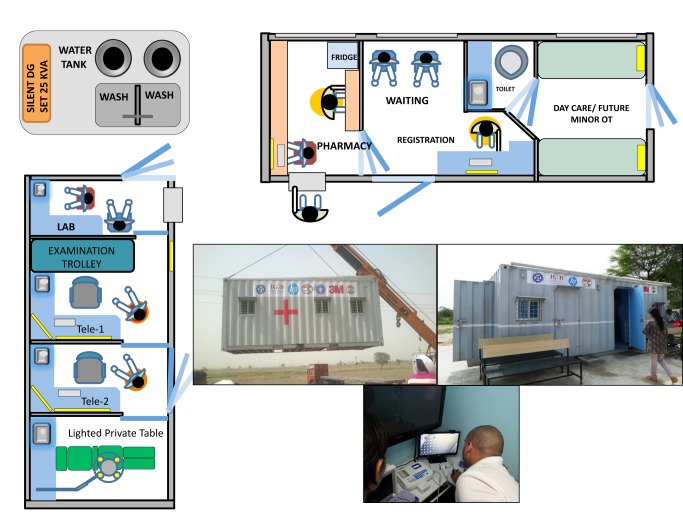
Layout of the eHC. Two 20×8 feet containers were used in the proof-of-concept eHC. The telemedicine container (left) has provision for a small laboratory. The second container (top) is used for registration and pharmacy and also has space for a future minor operation theatre. The telemedicine container can be used by itself for basic eHC operations. Pictures of the deployment, exterior, and interior of the telemedicine container are shown.

The eHC, capable of providing primary health care (PHC) services (Box 1), was pre-fabricated within two half-size (20 ft×8 ft) shipping containers, because half-container trucks can easily navigate hilly terrain or narrow roads [15]. The design (Figure 1) includes a registration area for the initial patient encounter and recording of vital signs, two air-conditioned telemedicine studios with provision for minor surgery or wound care, a lab area, and a pharmacy. Electrical generator sets are incorporated for self-sufficiency. Equipment available in the eHC has digital output, wherever possible; for example, for infant/adult scales, thermometers, automated blood pressure devices, pulse oximeters, electrocardiograms, spirometers, and glucometers. These components are connected to a mixed wired and wireless local network, forming a self-sufficient local health network (HN). Critical analog equipment use, such as for refrigerators, has been added to the HN by monitoring their electrical consumption. Access to eHC is via biometric login, intended to mitigate provider absenteeism.

**Box 1. Primary Health Care Services Available in an Integrated eHealth Center**

Medical Equipment

- Vitals
  - Digital thermometer, digital blood pressure, pulse-oximeter, scales
- Telemedicine
  - EMR, e-stethoscope, audio/video system, digital ECG, digital spirometer
- Clinical chemistry
  - Digital glucometer, complete blood count system
- Miscellaneous
  - Lighted examination/minor surgery table, ophthalmoscopes, oxygen concentrator, refrigerator
  - Ultrasound installation was planned but has been delayed due to prevalence of female foeticide. Radiography may be substituted.

Services

- Maternal child welfare
  - Growth monitoring, screening
- Screening and treatment for chronic diseases
  - Hypertension, diabetes, obstructive airway disease
- Treatment of acute non-critical illnesses
  - Uncomplicated infections, minor trauma, chronic obstructive pulmonary disease/asthma exacerbations
- Tele-consults and referrals, pharmacy services

### eWorkflow and Telemedicine

A modified version of the OpenEMR electronic medical record (EMR) system [16], running on a local server, receives clinical data and enables the clinical workflow. The eHC datastream and EMR are also connected to a remote health cloud via the nearest mobile phone tower. Telemedicine may be enabled between the eHC and any tertiary care hospitals via the health cloud. Other than audio and video connectivity between sites, cloud-based access to the EMR allows direct entry of orders and notes, as well as desktop sharing.

### Remote Monitoring and Analysis

A web-based dashboard (Figure S1) provides an overview of the operational status of the eHC, provision of health care, and critical alerts. EMR data, available in mySQL format, may be continually analyzed for data trends, such as number of new cases, diagnoses, demographic breakup, etc. Critical alerts can be set as per requirement, such as notification of very late arrival of the health care providers at the eHC. The dashboard integrates the health-data analysis, usage of various types of equipment, alerts, and a real-time view of the premises. This novel aspect of the eHC design can bring transparency to health care delivery and is a potential game changer.

## Implementing the eHC Solution

### Deployment

The first eHC was deployed, with requisite permissions from state health authorities, in Village Chausala, District Kaithal, Haryana, India. We surveyed the villages in the constituency of Naveen Jindal, a member of parliament, who has championed the eHC. Population size, distance to the nearest functional health care center, road connectivity, basic infrastructure, and keenness of the village leadership towards installing an eHC, were key aspects of the survey. Chausala is a small village of about 7,000 people that, at the time of our survey, did not have a functional primary health center requiring people to travel about 8 km for access to adequate health services. Electricity was available but only for a few hours a day, water was abundant, and road connectivity was suitable for a truck that could transport the eHC to the village site. The work required at the chosen site prior to deployment included construction of a septic tank and a concrete base on which the eHC would be set down. Construction was facilitated by the village leadership and took about 1 month, as did the fabrication and fitting of the eHC, which was then shipped by truck and deployed by a crane over a period of 2 days. Creating appropriate electrical, water, and waste disposal connections took another 2 days. Photographs of the process and site are shown in Figure 1.

### Operationalizing

While the eHC is designed to be operable by trained non-medical manpower under telemedicine supervision, we chose medical interns who had completed the graduate level curriculum of Bachelors in Medicine and Bachelors in Surgery (MBBS) course, and nursing students from Maharaj Agrasen Medical College (MAMC) to complete elective rotations at the eHC as a part of government -mandated compulsory rural service as well as educational exposure to emerging fields like telemedicine. Interns have temporary medical practice licenses from the Medical Council of India or State Medical Councils and undergo compulsory training in community medicine as part of the medical curriculum. Specialist faculty at MAMC were available via telemedicine at designated times. One registered nurse from the local area is the only permanent staff member, in addition to the interns and students on rotation. Specialist consultations are exclusively by telemedicine. Complex cases requiring in-person examination or detailed hematology workup are referred to the nearest suitable medical facility; however, such cases are few. Most importantly, digital data are being recorded at each encounter, including tests and biometrics, and then uploaded to secure servers. Repeat visits have been smooth with all patient data readily available. Given that even most tertiary hospitals of India do not have EMRs, their use in the eHC is a major step.

### Performance

The patient response has been enthusiastic and about 40 patients are seen daily. Table 1 shows utilization of the eHC over 5 months based on verifiable biometric registration data recorded on the EMR. The age-gender distribution of 3677 patient visits is further stratified by repeat visits. While 55% of the visits were by women and girls, there is a gender skew against girls younger than 18 yrs (p<0.001) in eHC visits that exceeds the general gender skew (1140∶1000 M∶F, http://censusindia.gov.in/2011-prov-results). The patient population is representative of the region.

**Table 1:** Details of Utilization of the eHealth Center.

	Male		Female		p-value
	n	%^a^	n	%	
**Total subjects**	1659		2018		
0–5 yrs	102	6.1	73	3.6	<0.01
6–18 yrs	339	20.3	224	11	<0.001
19–60 yrs	856	51.2	1340	65.9	<0.001
>60 yrs	362	21.7	381	18.8	NS

Repeat visitsAny repeat visit (>5 visits)		Repeat visit %^b^		Repeat visit %	
0–5 yrs	42 (1)	41.1	35 (2)	47.9	NS
6–18 yrs	179 (3)	52.8	112 (3)	50	NS
19–60 yrs	451 (9)	52.7	691 (19)	51.6	NS
>60 yrs	210 (12)	58	229 (22)	60.1	NS

Number of registrations and visits are stratified by gender, age, and frequency of repeat visits.

a Percentage of total subjects of indicated gender for each age group.

b Percentage of total subjects of indicated gender and age-group with repeat visits.

While most patient registrations are from Chausala village, with about a quarter of the population having already made at least one visit, we have seen an increasing widening of the catchment area, with many people from other areas preferring to come to the eHC than to access PHC in their own village, accounting for about a third of the visits. This preference to visit the eHC is a sign of it meeting patient expectations, but the resulting increased workload has led to quality concerns. Repeat visits, another indicator of performance, were frequent despite a short operational period. More than 50% of patients had at least one repeat visit, with about 5% of elderly (>60 yrs) patients having more than five visits.

The most common diagnoses are shown in the dashboard snapshot and positive diagnoses are further stratified by age and gender (Figure S1). Other than flu-like illnesses and non-specific dyspepsia, fungal skin infections, scabies, and probable allergic rashes account for a large number of patient visits. Muscle or joint pain is reported at early ages, possibly related to a very physical lifestyle. Most commonly prescribed medications, based on EMR data, expectedly fall in the classes of antacids, analgesics/antipyretics, and anti-histamines. Relatively few antibiotic prescriptions for the common cold have been recorded, which is a good performance indicator, given the tendency for indiscriminate overuse. Notably, because there had been no functional PHC within an 8-km radius of this village before the deployment of the eHC, most villagers visited doctors only for critical issues or when minor health troubles became major problems. When the eHC opened, we saw many patients with easily treatable skin conditions, such as scabies or fungal infections, who had not bothered to see a doctor previously because of the effort required. Patient satisfaction and good treatment response for such reversible conditions made people living in the village more aware and sensitive to their health and well-being.

## Challenges and Lessons Learnt

Our primary challenge with the eHC in Chausala is maintaining the supply chains for all essentials and ensuring timely attendance of health care personnel, despite being able to e-detect absenteeism. Another ongoing challenge is encouraging complete documentation within the EMR rather than abbreviated visit summaries and incomplete studies. The dashboard has been very effective in letting us know of non-use of advanced equipment, downtimes, and short working days of the staff, which are being suitably addressed.

Within these limitations, the proof-of-concept eHC has shown it is a workable solution for rapidly providing basic health care at the doorsteps of people who have previously lacked access, while collecting at least some data relevant to research and policymaking. Quality assurance measures, including e-review of charts and discussion with outgoing interns, suggest that high-quality preventive health services and associated screening tests are difficult to accomplish in the face of very high demand. However, medical services provided at the eHC are better than those the villagers have received to date, based on direct feedback. Also, the dashboard feature permits transparent monitoring and corrections, where possible Deployment of more eHCs will prevent overloading of a few, as well as help to develop the benefits of scale. In order to permit greater use of eHCs in villages with poor electricity and in remote locations with an erratic supply of diesel, options for local renewable power generation are being pursued [17].

## The Road Ahead

A second eHC has been installed in Lakhimpur Kheri, Uttar Pradesh, using Council of Scientific and Industrial Research (CSIR) funds supplemented by Hewlett Packard, India (HP). While CSIR and HP remain committed to providing internal support for further development and testing of the eHC solution, additional funds are expected from the National Rural Health Mission and from government-mandated corporate social responsibility (CSR) spending, which is particularly well-suited to the eHC solution because it demands proof of services provided. These funds will be used to create not only more eHCs but also standardized operational protocols, referral systems, and monitoring, such that provision of health care and collection of health data are quality-assured.

To address the need for trained manpower, we anticipate that the expected short Bachelor of Rural Medicine and Surgery (BRMS) degree program, or similar, will create a new cadre of rural doctors and medical assistants [18], many of whom may be attracted to working in eHCs to gain wider exposure to the state-of-art in medicine. Providing training to paramedical and non-medical staff for effective use of telemedicine will be the parallel approach. We are also investigating crowd-sourcing solutions to increase the specialist physician pool for complex cases. One solution may be a portal where physicians can volunteer time and be connected to an eHC in need of expertise.

In addition to the eHC affording creation of a health care infrastructure in rural settings, its compactness makes it suitable for use in urban slums, an option that is being explored. Other ongoing work includes development of automated analyses and systems that can continuously sift through data; identify anomalies or critical values such as a spike in the number of fevers or a very high glucose reading; and take appropriate actions such as to warn the medical staff of an epidemic or to notify the physician (or patient) by mobile-phone message. While these targets are ambitious, none are beyond today's technology.

## Supporting Information

Figure S1 **The eHC Dashboard.** A web-based dashboard continually updates the operational status of the eHC and provides an overview of the health services provided (A). A snapshot from March 2013 shows alerts regarding operational problems that were rectified. A further break up of the major disease types shown in the dashboard, by age and diagnoses, is also shown (B). Flu-like illnesses accounted for the most visits. Dermatological illnesses were surprisingly high, as were arthritic/musculoskeletal conditions.
(TIF)

## Abbreviations

eHC: integrated eHealth Center
EMR: electronic medical record
HN: health network
MAMC: Maharaj Agrasen Medical College
PHC: primary health care
