# Supplementary figures and images for: Integrating Health Care Delivery and Data Collection in Rural India Using a Rapidly Deployable eHealth Center
Source: PLoS Med. 2013 Jun 25;10(6):e1001468. doi: 10.1371/journal.pmed.1001468 (PMC3692411; doi:10.1371/journal.pmed.1001468)

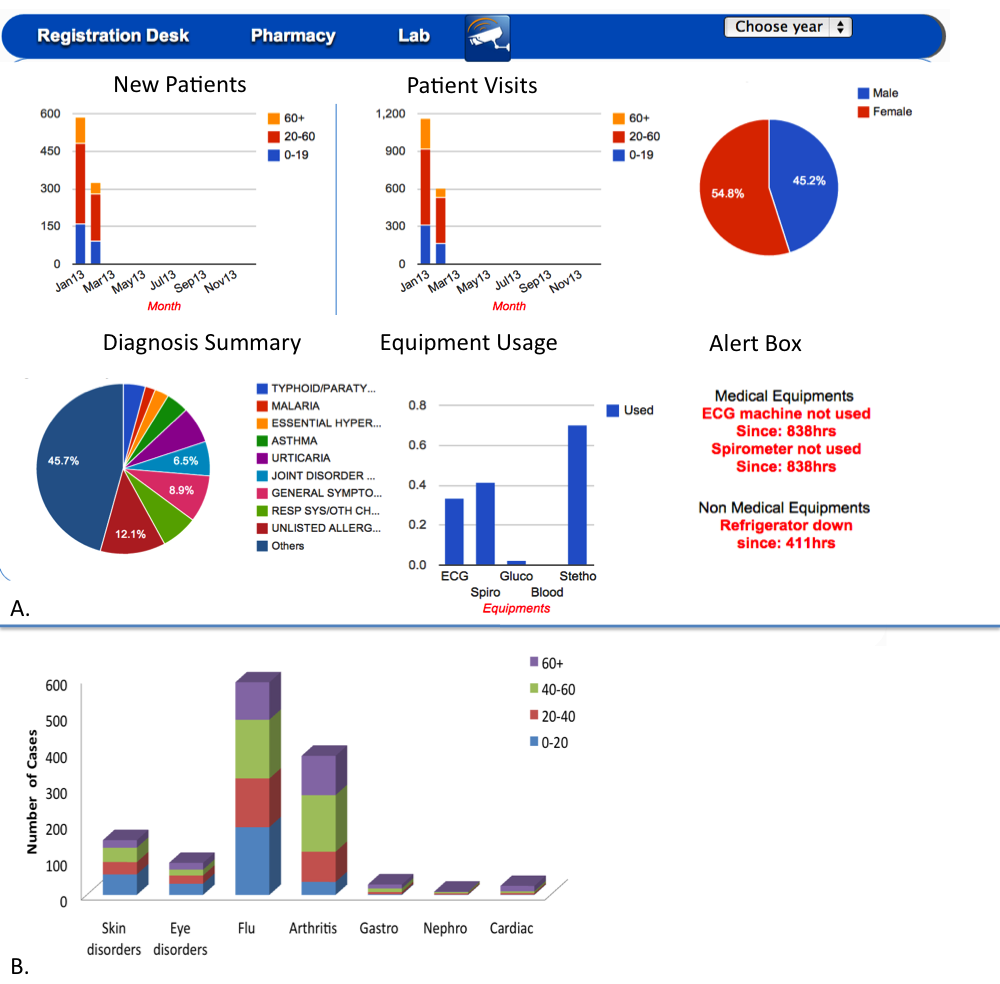

Supplement: Figure S1 — The eHC Dashboard. A web-based dashboard continually updates the operational status of the eHC and provides an overview of the health services provided (A). A snapshot from March 2013 shows alerts regarding operational problems that were rectified. A further break up of the major disease types shown in the dashboard, by age and diagnoses, is also shown (B). Flu-like illnesses accounted for the most visits. Dermatological illnesses were surprisingly high, as were arthritic/musculoskeletal conditions. (TIF) [file pmed.1001468.s001.tif]
